# Colonic Leiomyoma: A Case Report of an Uncommon Endoscopic Finding

**DOI:** 10.7759/cureus.50879

**Published:** 2023-12-21

**Authors:** Cristina Silva, José Mendes, Amélia Gaspar, Filipe Leal, Rui Almeida

**Affiliations:** 1 Unidades de Saúde Familiar Mondego, Agrupamento de Centros de Saúde do Baixo Mondego, Coimbra, PRT; 2 Pathology, Centro Hospitalar Universitário de Coimbra, Coimbra, PRT

**Keywords:** abdominal pain, endoscopic mucosal resection, colonoscopy, polyps, leiomyoma

## Abstract

Leiomyomas are rare, benign tumors composed of smooth muscle cells. When found in the colon, they account for only about 3% of gastrointestinal leiomyomas. Typically asymptomatic, they are often incidentally discovered during endoscopic evaluations. This report describes the case of a 71-year-old female with abdominal pain and distension, whose colonoscopy revealed a 7 mm sessile polyp in the sigmoid colon. Histological analysis confirmed it to be a submucosal leiomyoma. Although endoscopic resection is a common therapeutic strategy, surgical treatment may be necessary for larger tumors or when malignancy is suspected. This case emphasizes the limitations of polyp classification through endoscopy alone and the important role histopathological analysis continues to play in this regard. Accurately diagnosing these lesions is crucial for appropriate treatment and surveillance, namely, in the primary healthcare context. Further research is needed to improve diagnostic capabilities and reduce the need for repeat colonoscopies, reducing patient burden.

## Introduction

Leiomyomas are benign tumors that are composed of well-differentiated smooth muscle cells, which can arise from the muscularis mucosa, muscularis propria, or vascular smooth muscle. While they can occur throughout the entire digestive tract, they are more commonly found in the stomach and small intestine [1,2]. Leiomyomas are very rare in the large intestine, accounting for only about 3% of all gastrointestinal leiomyomas. In these cases, they are more frequently seen in the left colon. Colonic leiomyomas are often small, asymptomatic, and discovered incidentally during routine endoscopic evaluations [3,4]. However, larger lesions can sometimes cause symptoms such as abdominal pain, intestinal obstruction, rectal bleeding, and even perforations [1,2]. Many colonic leiomyomas are frequently misdiagnosed as mucosal polyps because they can present as solitary sessile intramural or intraluminal polyps. Therefore, histopathological analysis plays an important role in confirming the diagnosis and differentiating them from different types of polyps [1,2,5].

We present a case of a 7 mm sessile sigmoid colon polyp that was resected and later found to be a leiomyoma on histological examination.

## Case presentation

A 71-year-old female presented to her family doctor with dull, constant pain in the left hemiabdomen, particularly in the flank. The pain was worse in the sitting position and radiated to the left lumbar region. Abdominal distension was also reported but without changes in bowel movements or stool characteristics, genitourinary symptoms, nausea, vomiting, or weight loss. The symptoms had been ongoing for several months, but she was unsure of the exact duration. Physical examination was unremarkable. She had undergone a colonoscopy for colorectal cancer screening 10 years earlier, with no abnormal findings.

**Figure 1:**
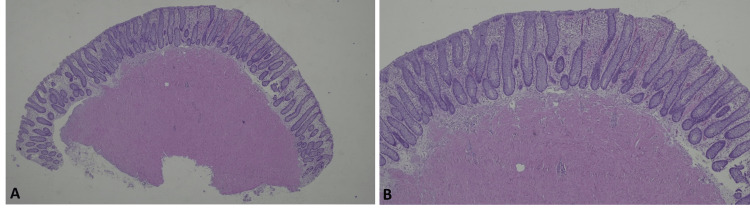
Histological images (hematoxylin and eosin staining). On the surface, there is well-preserved colon mucosa (A and B: ×40 and ×100, respectively).

**Figure 2:**
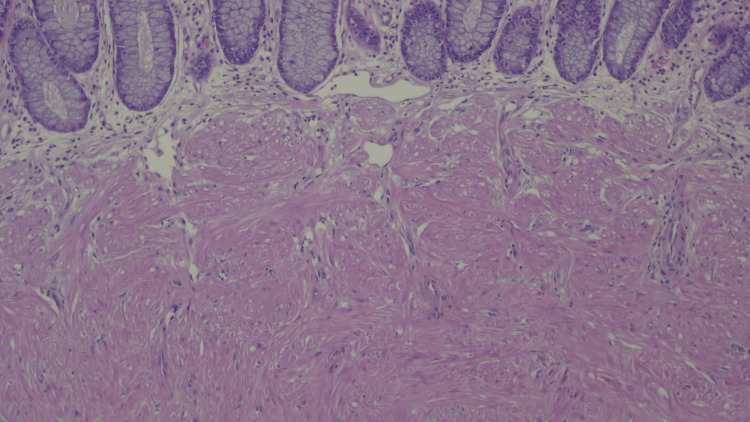
Submucosal lesion with well-defined border and fascicles of spindled cells with eosinophilic cytoplasm and bland nuclei, without mitotic activity (×200).

As a new colonoscopy was due as part of routine colon cancer screening, we took the opportunity to recommend it right away, but the patient was unable to complete the procedure due to a vasovagal syncope during the preparation. An abdominal ultrasound was also performed which described a focal parietal thickening of the sigmoid colon that was difficult to evaluate via ultrasound by the radiologist. A colonoscopy was, once again, recommended and performed (Boston Bowel Preparation Scale 9/9). It revealed a 7 mm sessile polyp at the 40 cm mark. After removal, a small hemorrhage was controlled with an endoscopic hemoclip. Histological examination confirmed the polyp to be a submucosal leiomyoma (Figures 1, 2).

Although it was clinically unlikely that the excised polyp was responsible for the patient’s symptoms, interestingly, the patient did not report any further complaints.

## Discussion

This case highlights the challenges of identifying the nature of a polyp exclusively through its endoscopic characteristics. It is particularly relevant when making the distinction between different types of polyps, which are divided into polypoid (type I) and non-polypoid (type II and III) according to the Paris classification, a morphological classification system of superficial mucosal lesions that can be used for endoscopic polyp evaluation. By classifying polyps into different types, it is also possible to assess the probability of a polyp being cancerous.

A majority of gastrointestinal polyps have an epithelial origin. In the colon, most lesions are adenomas (mostly tubulo/tubulo-villous with low-grade dysplasia). Nevertheless, benign proliferations of nonepithelial cells such as those from underlying smooth muscle layers, known as leiomyomas, can arise in the colon and are typically found incidentally [4]. Small sessile polyps have been reported as the most common presentation of colonic leiomyomas; however, there have also been reports of leiomyomas of the colon presenting as firm, well-circumscribed, intraluminal, semi-pedunculated, or pedunculated polyps [6]. Endoscopists ought to keep in mind submucosal lesions such as leiomyomas and carcinoids as differential diagnoses while evaluating the surface features of left-sided colonic polyps.

A study performed by Miettinen et al. analyzed a series of 88 leiomyomas of the muscularis mucosae of the colon and rectum. The lesions were typically small and located predominantly in the rectum and sigmoid (72%). All tumors were composed of well-differentiated, eosinophilic smooth muscle cells that were seen immediately beneath the mucosa. They concluded that these tumors typically present as incidental intraluminal polyps endoscopically and are clinically harmless lesions [7].

Although a more widespread use of screening colonoscopies has allowed for an increased accuracy of leiomyoma endoscopic diagnosis, further research is required to improve the ability to distinguish them from more common polyps. When the lesions present as submucosal, an endoscopic ultrasound could be useful in determining therapeutic strategies, providing valuable information on the size and extension of the tumor [3]. Moreover, flat lesions may require the use of endoscopic mucosal resection or endoscopic submucosal dissection techniques [4]. Xu et al. studied 106 patients with gastrointestinal leiomyomas diagnosed through endoscopic ultrasonography (EUS) and concluded that EUS had a very important diagnostic value for gastrointestinal leiomyomas. The nature of the lesions was accurately ascertained in 54 of the 57 patients via EUS (94.7% diagnostic accuracy). EUS can clearly show the origin of gastrointestinal leiomyomas, either from the muscularis mucosae or the muscularis propria [8].

The therapeutic strategy of colonic leiomyomas can also change according to the endoscopic findings and may vary from simple endoscopic resections to elective surgical removal of the lesion in cases involving voluminous tumors, the presence of multiple lesions, or if malignancy (tumor size >5 cm, presence of mesenteric adenopathy) is suspected [6,9,10]. Complete resection with an adequate margin of tissue all around the polyp, such as in the present patient, is crucial to prevent repeat colonoscopies, reducing the financial burden and emotional distress for patients, which is important in the context of quaternary prevention.

Choi et al. [3] evaluated the efficacy and clinical outcomes of endoscopic removal for colorectal polypoid leiomyomas from 22 patients with polypoid leiomyomas arising from the muscularis mucosae who underwent endoscopic removal at a single-referral gastrointestinal endoscopy unit. Most polypoid leiomyomas were small, asymptomatic lesions (<1 cm) and located predominantly in the left colon. Ten leiomyomas were removed using cold biopsy forceps and 12 were resected by conventional polypectomy or endoscopic mucosal resection. No local remnant lesions were found in 19 patients who underwent at least one follow-up colonoscopy. Thus, the endoscopic removal of colonic leiomyomas is considered, in general, an appropriate treatment modality because most arise from the muscularis mucosa and it often ensures the entire tumor is removed. According to the available literature, these lesions are inherently benign indolent neoplasms, and the overall prognosis is good [2,6-10].

In this case report, there is an interesting detail concerning the abdominal ultrasound, which described a “focal parietal thickening of the sigmoid colon.” Increased wall thickness is the main finding in bowel studies and seems, at first glance, to be a very nonspecific sign, but it can represent inflammatory, infectious, ischaemic, or tumorous conditions. A careful examination of the thickened bowel segment during an ultrasound while keeping in mind the patient’s clinical manifestations can reduce the differential diagnosis [11]. An abdominal ultrasound can offer the opportunity to study the digestive tract in a non-invasive way, which is also readily available and has good tolerance; however, it is a procedure with a steep learning curve and depends on the experience of the radiologist. This type of finding should not be underestimated and further diagnostic investigation should be performed.

Endoscopically similar lesions may have different treatments, prognoses, and long-term follow-up recommendations and must be adequately diagnosed and distinguished from each other. The general practitioner, who is often the first line in health care, uses colonoscopy and histologic results as diagnostic tools in a wide range of circumstances, ranging from simple cancer screening to challenging cases, such as the one described here. As colonic leiomyomas are a very rare condition, it is important to report such cases to increase awareness among endoscopists and physicians.

## Conclusions

The case presented in this article underscores the challenges of accurately identifying the nature of a lesion based only on endoscopic characteristics. The distinction between different types of polyps is decisive for determining appropriate treatment strategies and follow-up recommendations. Further research is needed to establish solid criteria that would allow an increase in diagnostic power through endoscopic procedures and, consequently, more efficient treatment strategies. Although the definitive diagnosis is only achieved via histological analysis, early identification and proper management of these lesions during a colonoscopy can reduce the burden on patients and minimize the need for multiple procedures. Endoscopic polypectomies, minimally invasive and cost-effective procedures, seem to be an adequate treatment for small colonic leiomyomas, especially because, according to several articles, fully resected colonic leiomyomas do not recur.
